# Advances in Peptide-Functionalized Polymeric Nanocarriers for Cancer Therapy

**DOI:** 10.3390/pharmaceutics18101231

**Published:** 2026-09-28

**Authors:** Ojaswitha Ommi, Obdulia Covarrubias-Zambrano, Stefan H Bossmann, Divya Kamath

**Affiliations:** 1 Department of Cancer Biology, University of Kansas Medical Center, Kansas City, KS 66160, USA; ocovarrubias@kumc.edu (O.C.-Z.); sbossmann@kumc.edu (S.H.B.); 2 Department of Chemistry and Biochemistry, The University of Toledo, Toledo, OH 43606, USA; 3 The University of Kansas Cancer Center, Kansas City, KS 66160, USA

**Keywords:** polymer, nanocarriers, targeting peptides, cell-penetrating peptides, tumor-microenvironment, therapeutic peptides, cancer

## Abstract

**Background/Objectives:** Peptide-functionalized polymeric nanocarriers are redefining targeted cancer therapy by combining the molecular specificity of peptides with the structural versatility of polymers. This review aims to highlight recent advances in peptide-functionalized polymeric nanocarrier systems designed to overcome key biological barriers to effective drug delivery. **Methods:** This review examines peptide-functionalized polymeric nanocarriers, including polymeric nanoparticles, micelles, polyplexes, and polymer–drug conjugates engineered with targeting, cell-penetrating, tumor-penetrating, and tumor microenvironment-responsive peptides. Emerging design strategies, including dual- and multi-peptide functionalization, stimuli-responsive linkers, and prodrug-like polymer architectures, are reviewed. **Results:** These peptide-functionalized polymeric nanocarrier systems have been investigated for the delivery of diverse therapeutic cargos, including small-molecule drugs, peptides, proteins, and nucleic acids. They demonstrate enhanced tumor localization, improved cellular internalization, and spatiotemporally controlled drug release, thereby contributing to significantly improved therapeutic efficacy and reduced systemic toxicity in preclinical studies. Advanced designs incorporating multiple peptide functionalities further enable precise modulation of drug delivery and therapeutic outcomes. **Conclusions:** Peptide-functionalized polymeric nanocarriers represent a versatile and powerful platform for next-generation cancer therapeutics. However, challenges related to stability, large-scale manufacturability, and clinical translation persist. Integration of data-driven approaches and artificial intelligence holds promise for accelerating optimization and facilitating clinical translation.

## 1. Introduction

Cancer is a major global health challenge, with millions of new diagnoses and deaths annually. Although conventional approaches such as chemotherapy [1], radiotherapy [2], surgery [3], and hormonal therapy [4], have improved substantially over recent decades, their clinical success is often limited by severe systemic toxicity, low selectivity towards tumor tissues, and development of therapeutic resistance [5,6,7,8]. Consequently, treatment outcomes remain suboptimal, highlighting the need for innovative therapeutic strategies that improve targeting precision while minimizing adverse effects [9,10].

Recent advances in nanomedicine mitigates these challenges through the development of nanocarriers whose nanoscale size, tunable surface properties, and ability to enhance pharmacokinetics improve drug delivery, stability, and tumor accumulation while reducing systemic toxicity through passive and active targeting strategies [11]. It also supports additional applications in diagnostics and regenerative medicine [12]. A wide range of nanocarriers including polymeric nanocarriers [13,14], micelles, liposomes [15], dendrimers [16], and inorganic systems (e.g., silica [17], gold [18], quantum dots [19]) have been developed for cancer diagnosis and therapy [20]. In addition, in silico approaches, including artificial intelligence (AI) and machine learning (ML), have facilitated the rational design of nanocarriers by predicting drug-carrier-protein interactions, self-assembly behavior, and drug-release profiles, thereby enabling improved targeting, stimuli responsiveness, and therapeutic performance [21,22]. One of the most important applications of nanocarriers is targeted and controlled drug delivery, enabling selective delivery of therapeutics to diseased tissues while minimizing systemic exposure [23]. Through mechanisms such as the enhanced permeability and retention (EPR) effect, stimuli-responsive release (e.g., pH [24], redox potential [25], enzymes [26], temperature [27], or external triggers including light, ultrasound and magnetic fields [28,29,30]), and ligand-mediated active targeting, nanocarriers enable site-specific drug release, and reduce off-target toxicity [31]. Despite their potential, the clinical translation of functional nanomaterials remains limited by challenges such as suboptimal biocompatibility, poor biodegradability, and uncertain long-term safety [32].

**Figure 1:**
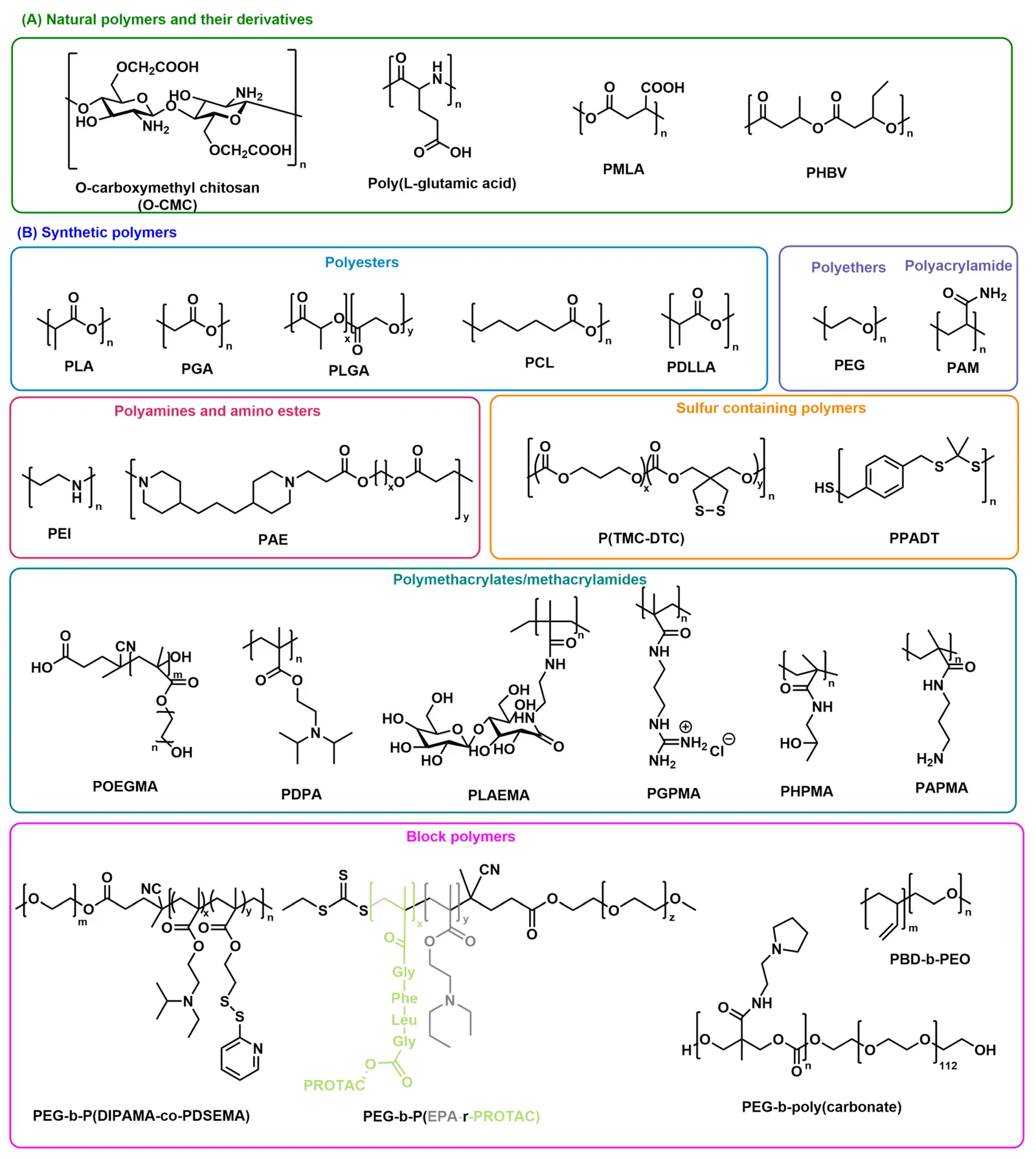
Structural representations of polymer families used in the peptide-functionalized nanocarrier systems. Polymers are classified according to their origin as (**A**) natural polymers and (**B**) synthetic polymers, and are further classified based on their predominant chemical structure and macromolecular architecture. PEG, Polyethylene glycol; PLA, Poly(lactic acid); PGA, Poly(glycolic acid); PCL, Poly(ε-caprolactone); PEI, Polyethylenimine; PMLA, Poly(β-L-malic acid); PDLLA, Poly(D, l-lactide); PAM, Polyacrylamide; PLGA, Poly(lactic-co-glycolic acid); PHBV, Poly(3-hydroxybutyrate-co-3-hydroxyvalerate); PPADT, Poly(1,4-phenyleneacetone dimethylene thioketal); PDPA, Poly(diisopropylaminoethyl Methacrylate); CMC, Carboxymethyl chitosan; PLAEMA, Poly(2-lactobionamidoethyl methacrylamide); PBD-b-PEO, Poly(butadiene)-b-Poly(ethylene oxide) where *b* denotes a block copolymer; PAE, Poly(β-amino ester); P(TMC-DTC), Poly(trimethylene carbonate-co-dithiolane trimethylene carbonate); PGPMA, Poly (3-guanidinopropyl methacrylamide); PHPMA, Poly(*N*-(2-hydroxypropyl) methacrylamide); PAPMA, Poly(3-Aminopropylmethacrylamide); POEGMA, Poly(oligoethylene glycol methacrylate; P(DIPAMA-co-PDSEMA), Poly(2-diisopropylaminoethyl methacrylate-co-pyridyl disulfide ethyl methacrylate);PEG-b-P(EPA-r-PROTAC), a block copolymer comprising pH-responsive tertiary amine methacrylate repeat units (EPA) and PROTAC-conjugated repeat units containing a cathepsin B-responsive Gly-Phe-Leu-Gly (GFLG) linker.

Within the nanocarrier systems, polymers play a central role due to their versatility and structural stability [33]. Polymers are macromolecules composed of repeating structural units that can be engineered to form stable and tunable drug delivery systems [34]. Polymer-based nanocarriers including polymeric nanoparticles [35,36,37,38], micelles [39], polyplexes [40], and certain polymer–drug [41] conjugates, offer advantages such as high structural integrity, efficient drug-loading capacity, multifunctional integration, prolonged circulation, reduced toxicity, and responsiveness to physiological stimuli [42,43,44]. For instance, cationic polymers can form complexes with negatively charged nucleic acids and undergo protonation/deprotonation within endosomes to disrupt membranes, ensuring cytosolic delivery before lysosomal degradation [45,46]. Environmental stimuli such as pH, temperature, redox potential, and ionic strength can modulate polymer solubility, conformation, and micellization [47]. These features have been exploited in cancer therapy to achieve controlled release under tumor microenvironment (TME) conditions or during externally applied hyperthermia [21,47]. The polymer families used in nanocarrier systems reviewed here incorporate a diverse range of natural and synthetic polymers, and structural representations of these polymers are shown in Figure 1. However, polymeric nanocarriers also present certain limitations, including limited cellular uptake and tumor penetration, burst release, insufficient target specificity, and reticuloendothelial system (RES) uptake [48,49].

To overcome these limitations and further enhance targeting precision, polymers are frequently functionalized with bioactive peptides that impart additional specificity and functionality. Peptides are short chains of amino acids linked to one another through covalent peptide bonds that possess inherent biological activity and molecular recognition capability [50]. Peptides can be broadly categorized into:

(i) Targeting peptides: These peptides enable receptor-mediated tumor recognition and uptake, reducing off-target effects.
(ii) Microenvironment-responsive peptides: These peptides are tumor microenvironment-responsive peptides (TME-peptides) and facilitate drug release in acidic or enzyme-rich TMEs, minimizing systemic toxicity.
(iii) Cell-penetrating peptides (CPPs): CPPs enable intracellular delivery of otherwise impermeable cargos.
(iv) Therapeutic peptides [51]: Therapeutic peptides possess inherent pharmacological activity and directly exert anticancer effects [52].
(v) However, peptides alone face limitations such as poor stability, proteolytic degradation, rapid clearance, and short in vivo half-life resulting in suboptimal biodistribution and pharmacokinetics [53].

Incorporation of peptides into polymeric nanocarriers to generate peptide-functionalized polymeric nanocarriers addresses many of these challenges by improving stability, prolonging circulation, and enabling multifunctional delivery, commonly accomplished through physical encapsulation by co-assembly, electrostatic interaction, and chemical bonding [44,54].

## 2. Peptide-Functionalized Polymeric Nanocarriers

### 2.1. Synthesis of Peptide-Functionalized Polymeric Nanocarriers

**Figure 2:**
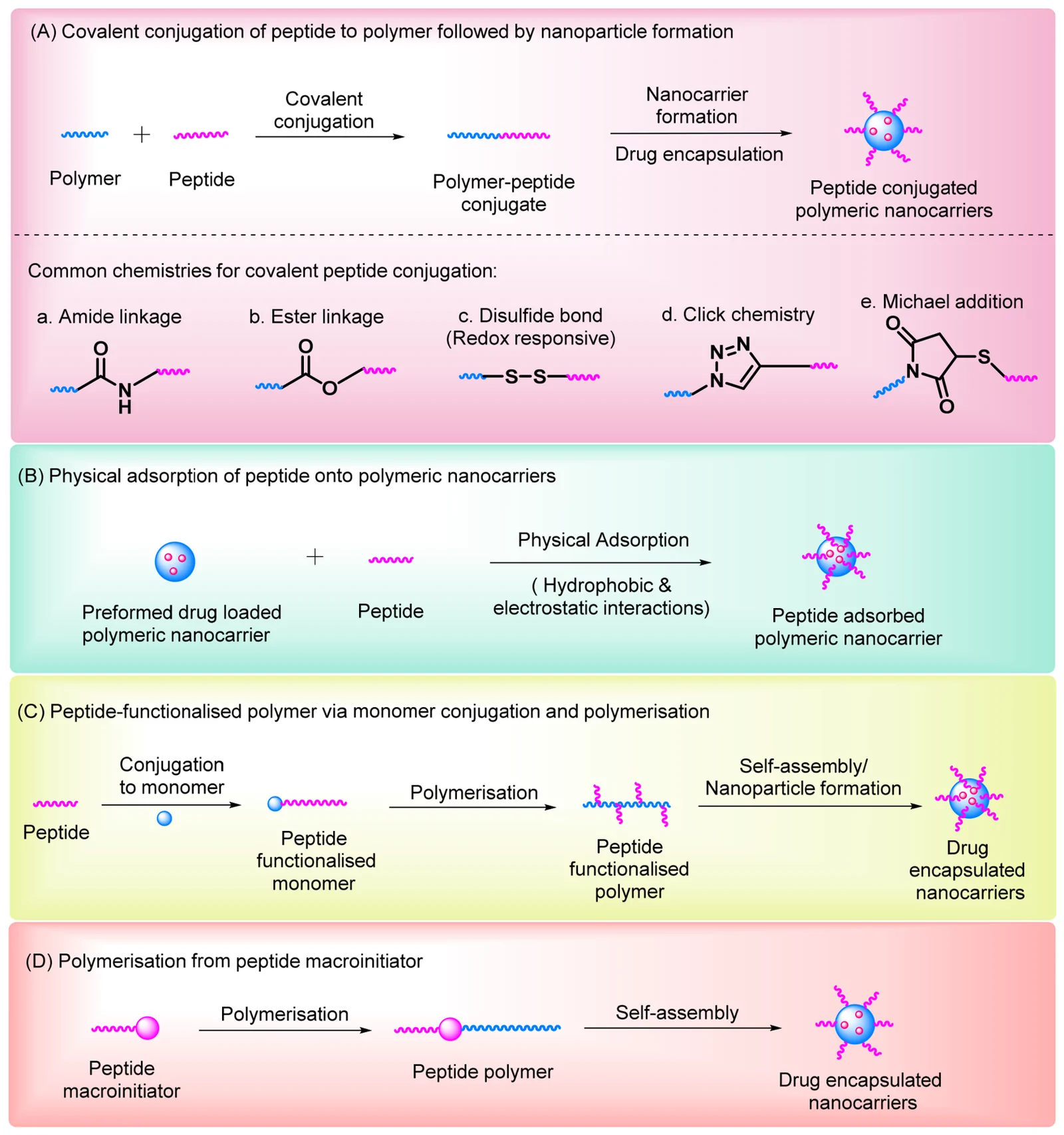
Peptide incorporation and conjugation strategies in polymeric nanocarriers include (**A**) covalent conjugation of peptide to polymers followed by nanoparticle formation, (**B**) physical adsorption of peptide onto preformed polymeric nanocarriers, (**C**) peptide functionalization of monomers followed by polymerization and nanoparticle formation, and (**D**) polymerization initiated from a peptide macroinitiator followed by self-assembly into nanocarriers.

The various strategies for peptide incorporation and conjugation within polymeric nanocarriers are illustrated in Figure 2. Intermolecular interactions including hydrogen bonding, electrostatic forces, π–π stacking, hydrophobic interactions, and metal coordination further stabilize peptide-functionalized nanoparticles enabling selective delivery of therapeutic agents.

(a) Covalent conjugation: Peptides can be incorporated into polymeric nanocarriers through covalent conjugation to polymers. This is typically achieved using:
  (i) Ester and amide linkages—cleavable through hydrolysis;
  (ii) Disulfide bonds—responsive to redox stimuli;
  (iii) Coumarin esters—responsive to light stimuli;
  (iv) Michael addition—Maleimide—thiol bonds;
  (v) Click chemistry.

Conjugation of the nanocarriers can be followed by the formation of drug-encapsulated nanoparticles [55].

(b) Physical adsorption: Peptides can be physically adsorbed to preformed polymeric nanocarriers through hydrophobic and electrostatic interactions.
(c) Polymerization: In some cases, peptide-functionalized polymers are synthesized by first conjugating the peptide to a monomer, followed by a polymerization process called Reversible Addition-Fragmentation chain Transfer or RAFT polymerization. Alternatively, polymerization can be initiated from a peptide macroinitiator to yield well-defined nanostructures [55].

Peptide-functionalized polymeric nanocarriers have been explored for the delivery of diverse therapeutic cargos, including small-molecule drugs, proteins, peptides, and nucleic acids (e.g., siRNA, miRNA, mRNA, and plasmid DNA) [56,57,58]. Because these therapeutic cargos differ in their physicochemical properties and biological targets, they encounter distinct delivery barriers [56]. For example, small-molecule therapeutics primarily benefit from increased half-life, tumor accumulation, and controlled or stimuli-responsive release to enhance therapeutic efficacy while minimizing off-target toxicity [59,60,61]. In contrast, nucleic acid therapeutics additionally require protection from nuclease degradation together with efficient intracellular delivery and endosomal escape [62,63,64]. Likewise, protein and peptide therapeutics present additional delivery challenges owing to their large molecular size, susceptibility to enzymatic degradation, limited cellular uptake, and the need to preserve structural integrity and biological activity during delivery [44,65,66,67].

This review highlights the potential of peptide-functionalized polymeric nanocarriers as targeted drug-delivery systems for cancer therapy, emphasizing the role of peptides in tumor targeting, cellular and tumor penetration, and stimuli-responsive or therapeutic drug release. Furthermore, polymer composition and its functional role, peptide conjugation strategies and linker design, including prodrug-like polymer linkers, are discussed. The reported nanocarriers are systematically classified based on peptide functionality, including targeting peptides, cell-penetrating peptides, tumor microenvironment-responsive peptides, dual-functional peptides, and therapeutic peptide-based delivery systems, with particular emphasis on specific peptide–polymer combinations and their therapeutic outcomes across different cancer types.

### 2.2. Targeting Peptide-Functionalized Polymeric Nanocarriers

Targeting peptides, or tumor-homing peptides, are short amino acid sequences engineered on nanoparticle surfaces to selectively bind targets (receptors or proteins) overexpressed on cancer cells or tumor-associated stromal and vascular cells. These peptides improve target-cell recognition and uptake of nanocarriers within the tumor microenvironment [68,69]. Targeting peptides are identified through integrated experimental and computational strategies to ensure high specificity and affinity for their intended targets [69]. Experimental methods include in vitro screening of synthetic peptide libraries against purified targets and in vivo techniques such as phage display and two-hybrid systems, which better mimic physiological conditions and enable enrichment of peptides with strong target binding capability. These methods are further complemented by in silico screening and molecular modeling [21], which predict binding interactions and accelerate peptide optimization prior to experimental validation [70]. Below we discuss key cellular targets that have been pursued for cancer therapy using the targeting peptide-functionalized polymeric nanocarrier.

#### 2.2.1. Transferrin Receptor (TfR)

The transferrin receptor (TfR) has emerged as attractive target owing to its iron-dependent overexpression in rapidly proliferating cancer cells [71,72]. Multiple TfR-targeted nanocarriers incorporating transferrin or transferrin-derived peptides have demonstrated enhanced tumor accumulation and antitumor efficacy compared with non-targeted formulations.

For example, the CBT-DC nanocarrier was designed with the TfR-targeting peptide T7(HAIYPRH) functionalized onto carboxymethyl chitosan/4-hydroxyphenylboronic acid pinacol ester for co-delivery of docetaxel (DTX) and curcumin (CUR). The CBT-DC nanoparticles leveraged TfR overexpression together with the acidic and ROS-rich tumor microenvironment to enable pH- and ROS-responsive co-delivery of docetaxel (DTX) and curcumin (CUR), effectively modulating the immunosuppressive tumor microenvironment and improving therapeutic performance in lung cancer models [73]. Similarly, transferrin peptide-modified Poly(lactic-co-glycolic acid)(PLGA) nanoparticles co-loaded with DTX and siRNA (siCCAT2) enhanced cellular uptake, overcoming multidrug resistance (MDR) in non-small cell lung cancer [74]. PEG-b-PCL-based polymersomes where Tf is conjugated to (poly(ethylene glycol)-b-poly(ε-caprolactone)) for delivery of doxorubicin (DOX) showed enhanced blood–brain barrier (BBB) transport, increased glioma cell uptake and improved therapeutic efficiency of doxorubicin (DOX) [75]. Collectively, TfR-targeted systems including T7-functionalized CBT-DC nanoparticles, Tf-modified PLGA nanoparticles and PEG-PCL polymersomes, demonstrated enhanced cellular uptake, superior antitumor efficacy, and where evaluated, greater tumor accumulation compared with non-targeted formulations and corresponding monotherapies [74].

Beyond direct ligand conjugation, indirect TfR-targeting strategies have also been explored. In one approach, a transferrin-binding peptide (TBP, CGGGHKYLRW) was covalently conjugated to disulfide-crosslinked poly(ethylene glycol)-block-poly(trimethylene carbonate-co-dithiolane trimethylene carbonate) mPEG-P(TMC-DTC) polymersomes, followed by non-covalent adsorption of transferrin onto the nanoparticle surface which enabled efficient delivery of doxorubicin (DOX) to TfR-overexpressing HCT-116 colorectal cancer (CRC) cells both in vitro and in vivo. These transferrin-decorated TBP polymersomes exhibited enhanced tumor accumulation and superior antitumor efficacy compared with non-targeted polymersomes and liposomal DOX [76].

#### 2.2.2. Cytokine and Chemokine Receptors

Cytokine and chemokine receptors such as CXCR4 are also overexpressed in more than 20 human cancers and play a key role in tumor progression and metastasis through interactions with stromal cell-derived factor-1 (SDF-1/CXCL12) [77,78]. Accordingly, CXCR4-antagonistic peptides have been widely investigated for targeted drug delivery and inhibition of metastatic signaling. Li et al. identified the CXCR4-antragonistic peptide E5 [79,80], and formulated it into 1,2-Distearoyl-sn-glycero-3-phosphoethanolamine (DSPE)-mPEG2000 nanomicelles via self-assembly. This improved the solubility and stability of the peptide, resulting in enhanced CXCR4 targeting and therapeutic efficacy when combined with DOX in acute myeloid leukemia models [81,82]. Similarly, polyethylene glycol-phosphatidylethanolamine (PEG-PE) micelles co-encapsulating E5 and DOX demonstrated enhanced binding affinity toward CXCR4-overexpressing solid tumor cells and more effective inhibition of CXCL12-mediated tumor migration compared to free E5 [83]. PEG-PE micelles also showed preferential lymph node accumulation and significantly improved antitumor efficacy in breast cancer models [84]. Likewise, CXCR4-antagonistic peptide LFC131 conjugated to O-carboxymethyl chitosan (O-CMC) nanoparticles enhanced receptor-mediated uptake and anticancer activity of DTX in CXCR4-overexpressing A549 lung cancer cells [85].

The AP peptide (CRKRLDRN) has been reported to exhibit high binding specificity toward interleukin-4 (IL-4) receptors overexpressing breast tumor cells and other pathological tissues. Leveraging this property, Wu et al. developed AP peptide-conjugated pH-responsive PEG-PLA/mPEG-PAE micelles. These micelles demonstrated enhanced tumor accumulation and improved therapeutic efficacy of DOX in MDA-MB-231 breast tumor-bearing mice, compared to free DOX and non-targeted micelles [86].

#### 2.2.3. Gastrin-Releasing Peptide Receptor (GRPR)

This receptor is overexpressed in several cancers [87,88,89] and exhibits high affinity for bombesin peptides, making them suitable targeting ligands [90,91,92]. Bombesin-conjugated PLGA nanoparticles loaded with DTX demonstrated significantly enhanced anticancer activity compared with free drugs and non-targeted nanoparticles in breast (MDA-MB-231) [93] and prostate cancer (PC3) cell models [94]. Similarly, bombesin-tethered reactive oxygen species (ROS)-responsive poly(ethylene glycol)-b-poly(1,4-phenyleneacetone dimethylene thioketal) (PEG-PPADT) micelles improved intracellular delivery and anticancer efficacy of monomethylauristatin F in prostate cancer therapy [95]. Likewise, octreotide-functionalized PTX-loaded PEG-b-PCL micelles targeted somatostatin receptor (SSTR)-overexpressing MCF-7 breast cancer cells, resulting in enhanced receptor-mediated cellular uptake and cytotoxicity compared with non-targeted micelles [96].

#### 2.2.4. Integrins

Another extensively studied targeting motif is the Arg-Gly-Asp (RGD) peptide, present in both linear and cyclic forms. RGD is widely used for integrin-mediated drug delivery due to the elevated expression of RGD-binding integrins in many cancers [97,98]. Multiple studies demonstrate that RGD functionalization of polymeric nanoparticles consistently enhances cellular uptake, antitumor efficacy, and reduces systemic toxicity compared with non-targeted formulations [69,96,99,100,101,102,103,104]. For instance, linear RGD-conjugated lecithin-PLGA nanoparticles co-loaded with paclitaxel (PTX) prodrug and cisplatin (CDDP) demonstrated enhanced cellular uptake, greater tumor accumulation, and improved therapeutic efficacy in lung cancer models [105]. Similarly, lipid–polymer hybrid nanoparticles (lpNPs) composed of PLGA-mPEG, RGD-PEG-cholesterol copolymers, and lipids (lecithin/cholesterol) and loaded with CUR achieved superior in vitro cytotoxicity and tumor growth inhibition against B16 melanoma cells, compared to free drugs and non-targeted formulations [106].

RGD functionalization has also been successfully integrated into stimulus-responsive systems. For example, RGD-functionalized PTX-loaded pH-responsive polydopamine-coated, poly(3-hydroxybutyrate-co-3-hydroxyvalerate) (PHBV) nanoparticles demonstrated enhanced therapeutic efficacy in hepatocellular carcinoma [107]. Likewise, redox-responsive PEG-PCL polymeric micelles co-delivering DTX and indocyanine green (ICG) enabled synergistic chemotherapy and photothermal therapy in 4T1 cells [108]. These findings demonstrate that RGD modification can be combined with stimuli-responsive platforms to enhance drug release, cellular uptake, and antitumor activity, highlighting the versatility of RGD across different carrier systems.

The biological activity of RGD peptides is strongly influenced by their three-dimensional structure. Cyclic RGD peptides exhibit distinct integrin-binding profiles, improved receptor affinity, minimal off-target interactions, and greater in vivo stability compared to linear counterparts [109]. Miura et al. showed that cRGD-linked poly(ethylene glycol)-b-poly(l-glutamic acid) micelles achieved more rapid and efficient transport across the blood–brain tumor barrier (BBTB), resulting in greater accumulation within glioblastoma tissue and improved therapeutic efficacy of 1,2-diaminocyclohexane)platinum(II) (DACHPt) in glioblastoma models compared to non-targeted cRAD-micelles [110]. Similarly, cRGDfK-functionalized PLGA nanoparticles selectively targeted α_v_β3 integrins-overexpressing SKOV-3 human ovarian cancer cells and enhanced gemcitabine (GEM) delivery even at low doses [111]. Zhu et al. further showed that cRGD-functionalized, DOX-loaded reduction-sensitive PEG-SS-PCL micelles maintained circulation stability while selectively exposing cRGD ligands within the reductive tumor microenvironment. This resulted in improved tumor accumulation, enhanced tumor growth inhibition and reduced systemic toxicity compared to non-targeted and reduction-insensitive controls [112]. RGD targeting has also been successfully extended to nucleic acid delivery. For example, cRGD-functionalized lpNPs composed of egg phosphatidylcholine, PEGylated lipids and a reduction-sensitive polyethyleneimine (PEI) derivative efficiently delivered survivin siRNA, resulting in enhanced cellular uptake, increased gene silencing efficiency and potent antitumor activity in HepG2 models, while maintaining minimal toxicity [113]. In addition, Demirgöz et al. developed PR_b, an α_5_β_1_ integrin-targeting peptide that mimics the fibronectin adhesion motif. PR_b conjugated poly(butadiene)_185_-b-poly-(ethylene oxide)_60_ (PBD_185_-b-PEO_60_) polymersomes enabled selective delivery of tumor necrosis factor-α (TNF-α) to prostate cancer cells. These polymersomes demonstrated enhanced cellular internalization and improved therapeutic efficacy compared with non-targeted carriers [114].

#### 2.2.5. Other Cellular Receptors

CD44 is another important tumor biomarker that is overexpressed in multiple cancers including breast, colon, and prostate. CD44 plays a key role in cancer stem cell-mediated tumor progression and represents an important therapeutic target [115]. CD44-targeting peptide-conjugated polymeric nanocarriers, including CD44 peptide-conjugated PEG-b-hydroxyethyl starch(HES)-b-PLA micelles delivering emodin [116] and A6 peptide-modified PCL-PEG nanoparticles delivering CUR [117], demonstrated significantly enhanced cellular uptake, improved cytotoxicity, and favorable safety profiles in CD44-expressing MDA-MB-231 breast cancer cells compared with non-targeted formulations. Similarly, epidermal growth factor receptor (EGFR)-targeting GE11 peptide-functionalized polymeric nanocarriers demonstrated improved receptor-specific delivery. GE11-modified salinomycin-loaded lpNPs composed of PLGA-DSPE-PEG exhibited enhanced cellular internalization and therapeutic efficacy in osteosarcoma models [118], whereas GE11-functionalized DTX-CUR prodrug-loaded PLGA-PEG nanoparticles combined GE11-mediated cellular targeting with the EPR effect and pH-responsive drug release to improve antitumor efficacy in prostate cancer models [119]. Tumor-homing peptides have also been successfully applied to overcome physiological barriers such as the BBB. Rabies virus glycoprotein (RVG) peptide-functionalized lpNPs enabled PTX delivery across the BBB by targeting nicotinic acetylcholine receptors (AChR) expressed on brain endothelial cells, thereby improving drug delivery to glioma-associated macrophages [120]. In addition, affibody- and neuropilin-targeting peptide systems have demonstrated improved receptor-specific delivery and therapeutic outcomes. Anti-HER2 affibody-functionalized N-(2-hydroxypropyl) methacrylamide-3-Aminopropylmethacrylamide (PHPMA- PAPMA) copolymers enhanced PTX delivery to HER2-overexpressing breast cancer cells [121], while iNGRt-modified DTX-loaded PCL nanoparticles enhanced neuropilin-1 (NRP-1)-mediated cellular internalization and improved antitumor efficacy in triple-negative breast cancer models [122]. These systems significantly reduced cytotoxicity, improved intracellular drug delivery, and therapeutic efficacy compared with non-targeted carriers. These findings further demonstrate the versatility of peptide-functionalized polymeric nanocarriers in targeting diverse tumor-associated receptors.

In addition to classical receptor-targeted strategies, tumor-homing peptides that recognize tumor-associated proteins and microenvironmental components have demonstrated significant potential for improving the selectivity and therapeutic efficacy of polymeric nanocarriers. For instance, Yakati et al. conjugated the CPKSNNGVC peptide onto PTX-loaded PLGA nanoparticles targeting monocarboxylate transporter 1 (MCT1) overexpressing CRC cells [123]. Similarly, Winer et al. conjugated the tumor-homing peptide F3 (CKDEPQRRSARLSAKPAPPKPEPKPKKPKKAPAKKK) to cisplatin-loaded polyacrylamide (PAA) nanoparticles to target angiogenic pathways in ovarian cancer [124].

Additionally, vascular-targeting peptides such as APRPG have demonstrated significant potential for improving tumor-specific drug delivery. Micelles formed from PEG-PLGA copolymers functionalized with the angiogenic vessel-targeting peptide APRPG (Ala-Pro-Arg-Pro-Gly) enabled selective delivery of PTX to tumor-associated endothelial cells. APRPG-mediated vascular targeting together with the long circulating PEG-PLGA micellar system, was associated with enhanced tumor accumulation and therapeutic efficacy, resulting in significantly greater tumor growth inhibition and prolonged survival in tumor-bearing models compared to non-targeted micelles and free drugs [125]. In another study, the vascular-targeting peptide K237 was conjugated to paclitaxel-loaded PEG-PLA nanoparticles to target vascular endothelial growth factor receptor-2 (VEGFR-2) expressed on angiogenic tumor endothelial cells, thereby enhancing anti-angiogenic and antitumor efficacy in a breast cancer xenograft model [126].

Conjugation of targeting peptides to polymeric nanocarriers enhances receptor-specific interactions, cellular internalization, and therapeutic efficacy [69]. Notably, most of the systems discussed here demonstrated high drug entrapment efficiency, elevated plasma concentrations and extended plasma half-life, and prolonged systemic circulation, all of which contribute to improved therapeutic performance. While targeting peptides enhance receptor-specific interactions after nanocarriers reach the tumor microenvironment, avoidance of nonspecific clearance remains a critical determinant of tumor delivery [127,128,129,130]. The representative peptide–polymer combinations discussed in this section, including their peptide sequences, polymer platforms, conjugation strategies, and targeted cancer types, are systematically summarized in Table 1.

### 2.3. Cell-Penetrating Peptide-Functionalized Polymeric Nanocarriers

While targeting peptides enhance tumor accumulation and receptor-specific binding, efficient intracellular delivery of therapeutic cargo remains a critical challenge, which can be addressed by employing cell-penetrating peptides (CPPs) [131]. CPPs are short, often cationic or amphipathic peptides capable of crossing cell membranes to deliver diverse therapeutic cargoes such as drugs, proteins, nucleic acids, or nanoparticles [132,133]. This intracellular delivery capability is particularly important for nucleic acid therapeutics, whose biological activity depends on efficient cytosolic delivery following endosomal escape [56,57,131]. They enter cells via energy-independent direct translocation mediated by interactions with membrane lipids or via energy-dependent endocytosis, with endosomal escape being a critical determinant of efficacy [134,135,136]. CPPs enhance intracellular delivery of therapeutics, but challenges include limited specificity, proteolytic instability, immunogenicity, and restricted tumor penetration [137]. Strategies such as tumor-activated CPPs, chemical modifications, or conjugation with target ligands or nanoparticles improve selectivity, stability, and therapeutic outcomes in cancer therapy [134,138]. CPP functionalization of polymeric nanocarriers has been shown to markedly enhance intracellular delivery and therapeutic efficacy, with outcomes depending on polymer type, CPP sequence, and stimulus responsiveness [131]. Below we summarize prominent CPP-functionalized nanocarriers along with their applications.

#### 2.3.1. Histidine-Rich Peptides

These peptides have been widely explored as endosomal escape-promoting motifs for nucleic acid delivery [136,139]. The pH-responsive buffering capacity of histidine residues facilitates endosomal destabilization and intracellular cargo release, thereby enhancing transfection efficiency. Accordingly, histidine-functionalized polymeric vectors such as PEI [140] and PAMAM [141] have demonstrated reduced toxicity and improved plasmid DNA transfection. Furthermore, Gu and colleagues developed pH-responsive carboxymethyl poly(L-histidine)-coated poly(β-amino ester) (PbAE)/DNA ternary complexes for gene delivery. The resulting nanoparticles (174 nm) exhibited reduced cytotoxicity and significantly enhanced transfection efficiency in both HEK293 and B16-F10 cells, as well as improved in vivo gene expression, compared with uncoated PbAE/DNA complexes [142].

#### 2.3.2. VIPER (Virus-Inspired Polymer for Endosomal Release)

Cheng and colleagues developed a block copolymer composed of a polycationic poly (OEGMA-co-DMAEMA) segment for nucleic acid condensation and a pH-responsive poly (DIPAMA-co-PDSEMA) segment functionalized with the membrane-lytic peptide melittin to promote trafficking to the cell cytosol. Under acidic endosomal conditions, exposure of melittin triggers membrane disruption and efficient endosomal escape, resulting in enhanced in vitro and in vivo plasmid DNA delivery (YOYO-1 DNA) [143]. In a subsequent study, the same group extended the application of VIPER to siRNA delivery, where the melittin-functionalized polymer efficiently condensed siRNA, promoted endosomal escape, and achieved potent gene silencing both in vitro and in vivo, resulting in >75% GAPDH knockdown in mouse lungs following pulmonary administration [144].

#### 2.3.3. HIV-1 Trans-Activator of Transcription (TAT) Peptide

The TAT peptide is one of the earliest and most extensively investigated CPPs and has been widely incorporated into polymeric and other pharmaceutical nanocarriers to facilitate the intracellular delivery of diverse therapeutic cargoes [135]. For example, PTX-loaded TAT-conjugated PLGA nanoparticles increased nanoparticle uptake and retention in mesenchymal stem cells, leading to robust tumor growth inhibition in an orthotopic lung cancer model [145]. Additionally, dual pH-sensitive TAT/PEG-DMMA(2,3-dimethylmaleic anhydride)-coated poly(β-L-malic acid)-polyethylenimine (PMLA-PEI) micelles enabled tumor-specific activation by exposing TAT in the acidic tumor microenvironment, achieving superior DOX release and reduced systemic toxicity in both in vitro and in vivo models [146]. Similarly, a dual-responsive mixed polymeric micelle composed of TAT-modified poly(ethylene glycol)-polyphosphoesters (TAT-PEG-PHEP) and 2-nitroimidazole-grafted PEGylated polyphosphoesters PEG-b-P (AEP-g-NI) employed pH-responsive TAT reactivation and hypoxia-triggered micelle dissociation to improve tumor-specific cellular uptake and controlled release of chlorin e6(Ce6) and tirapazamine(TPZ) in MCF-7 tumor models [147]. Extending this strategy, a pH-responsive polymeric micelle composed of PLA-b-PEG-b-poly(l-histidine)-TAT and poly(l-histidine)-b-PEG was designed to shield TAT during circulation and expose it under mildly acidic tumor pH, enhancing cellular uptake. Endosomal acidification further triggered micelle destabilization, rapid DOX release, and poly(histidine)-mediated endosomal escape, enabling efficient cytosolic and nuclear delivery. This resulted in 3.8–8.8 fold lower IC_50_ values than free DOX and significant tumor regression in xenograft models with minimal systemic toxicity [148].

TAT-conjugated PEG-PCL micelles carrying a reduction-sensitive camptothecin (CPT) dimer combined FRET-based drug release reporting with acidic tumor-triggered TAT activation, enhancing uptake, cytotoxicity, tumor accumulation, and growth inhibition while reducing systemic toxicity in MDA-MB-231 cells [149]. Similarly, Kanazawa et al. incorporated siRNA and CPT into TAT-functionalized mPEG-PCL micelles for intranasal delivery, achieving enhanced nose-to-brain transport, improved anti-glioblastoma efficacy, and prolonged survival in glioblastoma-bearing (C6 glioma) models [150].

(a) Octa-arginine (R8) peptide: PArg is another highly cationic CPP commonly employed to promote efficient cellular internalization through electrostatic interactions with negatively charged cell membrane components, particularly heparan sulfate proteoglycans, thereby enhancing intracellular cargo delivery [151,152,153]. Hyperbranched polymers based on Poly(di-isopropylaminoethyl Methacrylate) (hPDPA) containing adamantane groups, functionalized with β-cyclodextrin-linked PEG and PArg, consistently improved cytosolic access of DOX [154] or β-lapachone-cargo [155]. It also showed improved cellular internalization and apoptosis in HeLa cells compared to peptide-free hPDPA/PEG nanocarriers. Similarly, R8-conjugated PEGylated PAMAM dendrimers co-functionalized with vitamin E succinate significantly enhanced cellular internalization, tumor spheroid penetration, apoptosis, and paclitaxel-mediated antitumor efficacy in A549 lung adenocarcinoma cells, multicellular tumor spheroids, and B16-F10 melanoma-bearing mice [156].
(b) WTAS is a rationally designed CPP developed by modifying a microtubule-associated sequence (MTAS) peptide [157] to enhance intracellular delivery of therapeutic cargo [158,159]. WTAS has been employed in combination with anticancer peptides, such as SA-K6L9-AS, to generate the in vitro-transcribed mRNA-encoded hybrid construct WTAS-K6L9, enabling intracellular production of cytotoxic peptides while bypassing extracellular degradation and delivery barriers, thereby enhancing anticancer efficacy [160].
(c) In addition to conventional CPPs, selective cell-penetrating peptides (SCPPs) can combine cellular internalization with tumor-cell specificity. The lung cancer-selective CPP, RLWMRWYSPRTRAYGC was conjugated to PEG-P(TMC-DTC) and co-assembled with PEG-P(TMC-DTC)-PEI to form crosslinked polymersomes. Methotrexate (MTX)-loaded polymersomes showed preferential A549 cell uptake, enhanced tumor penetration and accumulation, and improved antitumor efficacy compared with non-targeted polymersomes and free MTX [161].

Several other CPPs have also shown considerable promise. For instance, the well-investigated CPP penetratin was conjugated to chitosan–linoleic acid polymeric micelles, in which linoleic acid facilitated micelle formation and plasmid DNA condensation, while penetratin enhanced cellular uptake, nuclear localization, and transfection efficiency [162]. Similarly, Angiopep-2-conjugated PEG-PCL nanoparticles loaded with PTX enhanced blood–brain barrier penetration, and low-density lipoprotein receptor-related protein (LRP)-mediated glioma targeting, resulting in improved antitumor efficacy in glioblastoma models [163]. Also, lactoferrin-derived CPP-functionalized biodegradable PLGA nanoparticles enabled efficient plasmid DNA delivery by enhancing cellular uptake while exhibiting minimal cytotoxicity in two lung epithelial cell lines [164]. Collectively, these findings demonstrate that polymer–CPP conjugation significantly enhances intracellular delivery, endosomal escape, and therapeutic efficacy, with performance depending on the CPP sequence, polymer design, and stimulus responsiveness. The representative CPP-functionalized polymeric nanocarriers are summarized in Table 2.

## 3. Tumor-Penetrating and Targeting Peptide iRGD-Functionalized Polymeric Nanocarriers

The tumor-penetrating peptide iRGD (CRGDKGPDC) is a cyclic nine-amino-acid peptide containing an Arg-Gly-Asp (RGD) motif that enables selective targeting and deep penetration into tumor tissues [165,166,167]. Initially, iRGD binds to αvβ3 and αvβ5 integrins overexpressed on tumor vasculature, facilitating tumor homing, followed by proteolytic cleavage that exposes a cryptic C-end Rule (CendR) motif [168]. The exposed CendR motif subsequently binds to neuropilin-1 (NRP-1), triggering NRP-1-mediated endocytosis and transcytosis, thereby enhancing vascular permeability and promoting extensive penetration into the tumor parenchyma [169]. Unlike conventional RGD peptides that primarily localize near tumor vessels, iRGD enables efficient transport of conjugated or co-administered therapeutics deep within tumors through NRP-1-mediated transcytosis and a bystander transport mechanism [165,170,171]. Owing to its dual receptor targeting, low toxicity, ease of synthesis, and ability to improve intratumoral drug accumulation and therapeutic efficacy, iRGD has emerged as a highly promising targeting and penetration ligand for advanced cancer drug delivery systems [172]. Various studies have shown that iRGD functionalization enhances tumor penetration and therapeutic efficacy across diverse polymeric nanocarriers and cancer models. For instance, redox-responsive prodrug polymeric micelles composed of CPT-PEG-iRGD and co-loaded with the photosensitizer IR780 exhibited efficient BBB penetration, increased uptake in U87 glioma cells, and superior chemo-photodynamic antitumor efficacy [173]. iRGD-modified PTX-loaded pH-sensitive Poly(oligoethylene glycol methacrylate)-poly(2-(diisopropylamino)ethyl methacrylate) POEGMA-PDPA polymersomes showed preferential internalization in NRP-1-expressing gastric (MKN-45P) and colon (CT26) tumors, resulting in improved tumor selectivity and antitumor activity [174]. Similarly, iRGD-conjugated lipid–polymer hybrid nanoparticles composed of a PLGA core and a lipid shell of egg phosphatidylcholine, cholesterol, and maleimide-functionalized PEG-DSPE enhanced PTX and tetrandrine delivery in multidrug-resistant ovarian cancer by increasing cellular uptake, inhibiting P-gp, promoting ROS generation, apoptosis, and cell cycle arrest [175].

In pancreatic cancer, iRGD-functionalized PEG-b-poly(carbonate) nanoparticles with pyrrolodine sidechains delivering ERK inhibitor SCH772984 achieved synergistic cytotoxicity with GEM and prolonged survival in K-Ras-driven models outperforming either monotherapy [176]. Likewise, iRGD-modified PEG-b-poly(carbonate) nanoparticles co-loaded with GEM and GDC-0449 enhanced cellular uptake, tumor accumulation, and selective inhibition of pancreatic cancer proliferation [177]. These studies demonstrate that different polymer–iRGD combinations enable effective targeting and treatment of diverse cancer types, highlighting the versatility and broad therapeutic potential of iRGD-functionalized polymeric nanocarriers. Key examples of iRGD-functionalized polymeric nanocarriers and their tumor penetration capabilities are summarized in Table 3.

## 4. Tumor Microenvironment (TME)-Responsive Peptide-Functionalized Polymeric Nanocarriers

The tumor microenvironment (TME) represents an intricate network of biochemical and biophysical stimuli, including acidic pH, overexpressed enzymes, hypoxia, and redox imbalances, that differ significantly from normal tissues [178]. These distinctive features offer promising opportunities for the development of TME-responsive therapeutic strategies [178,179,180]. In this regard, integration of TME-responsive peptides onto polymeric nanocarriers has emerged as a highly effective approach to confer stimulus sensitivity while maintaining the structural stability and drug-loading capacity of polymer-based delivery systems. By serving as enzyme-cleavable linkers, stimulus-responsive segments, or functional motifs within the polymer architecture, these peptides enable site-specific activation of nanocarriers, resulting in controlled drug release, enhanced tumor penetration, and improved cellular internalization [181,182]. Consequently, TME-responsive peptide-functionalized polymeric nanocarriers represent a promising strategy to overcome key limitations of conventional cancer therapies, including poor tumor selectivity, systemic toxicity, and therapeutic resistance.

### 4.1. Single-Stimuli-Responsive Peptide-Functionalized Nanocarriers

These micelles have demonstrated significant potential for improving tumor-selective drug delivery. For instance, PEG-*b*-poly(D,l-lactide) (PEG-PDLLA) block copolymer micelles incorporating a matrix metalloproteinase (MMP)-responsive GPLGVRGDG peptide within the polymer backbone were synthesized via peptide-initiated polymerization. The resulting PTX-loaded micelles enabled protease-triggered activation, leading to prolonged circulation, enhanced tumor accumulation and antitumor activity in H22 tumor models [183]. In comparison, PEG-cholesterol micelles functionalized with an MMP-2 cleavable PLGVRK peptide linker and a prostate-specific membrane antigen (PSMA) targeting ligand, 2-[3-(1,3-dicarboxypropyl)ureido]pentanedioic acid (DUPA), combined enzyme responsiveness with receptor-specific targeting, achieving higher drug loading and more selective cabazitaxel (CAB) delivery to prostate tumors [184].

Stimuli-responsive exposure of penetrating motifs has also been demonstrated in PEG-GPLGVRG-PCL triblock copolymer vesicles incorporating an MMP-cleavable GPLGVRG sequence, within the polymer architecture. In this system, enzymatic cleavage selectively exposed poly (3-guanidinopropyl methacrylamide) (PGPMA) segments that mimic cell-penetrating peptides, thereby enhancing intracellular delivery and cytotoxicity of colchicine and marimastat in HT1080 fibrosarcoma cells. This system also suppressed primary tumor growth, prevented postsurgical recurrence, and inhibited metastasis by targeting both tumor cells and the tumor microenvironment in 4T1 murine breast cancer orthotopic tumor and postsurgical recurrence models [185]. Similarly, MMP-2/9-responsive polymer micelles incorporating an MMP-cleavable GPVGLIGK peptide linker between PEG and α-tocopherol succinate (mPEG–GPVGLIGK–α-TOS) further enhanced therapeutic performance by enabling selective drug release in enzyme-rich tumor environments (MMP-overexpressing HT1080 cells), resulting in improved cellular uptake, greater tumor accumulation, and superior antitumor efficacy of DTX compared to non-cleavable controls and free drugs [186].

Cathepsin K-responsive HPMA copolymer conjugates incorporating cleavable GGP-Nle tetrapeptide, bone-targeting alendronate and the anti-angiogenic agent TNP-470 demonstrated improved tumor localization, selective anti-angiogenic activity, and prolonged survival in osteosarcoma models [187]. Likewise, cathepsin B-cleavable PEGylated dendrimer–GEM conjugates using GFLG tetrapeptide enabled rapid enzyme-triggered drug release, resulting in significantly greater tumor growth inhibition compared to free drug treatment in 4T1 murine breast cancer model [188]. Similarly, PTX conjugated to poly(OEGMA) via the cathepsin B-cleavable GFLG peptide linker, formed enzyme-responsive polymeric nanoparticles capable of co-delivering the photosensitizer chlorin e6 enabling selective protease-triggered drug release and enhanced therapeutic efficacy through combined chemotherapy and photodynamic therapy in bladder cancer T24 cells and PDX models [189].

In comparison, cathepsin B-responsive branched glycopolymer nanoparticles based on the poly(2-lactobionamidoethyl methacrylamide) (PLAEMA) backbone and the same cleavable Gly–Phe–Leu–Gly peptide linker enabled controlled paclitaxel release while co-delivering the AKT inhibitor capivasertib, resulting in enhanced cytotoxicity, apoptosis, prolonged circulation, and significantly improved tumor growth inhibition compared to free drug combinations in gastric cancer models [190].

### 4.2. Dual-Stimuli-Responsive Peptide-Functionalized Nanocarriers

Polymersomes have a vesicular architecture and the ability to integrate multiple stimulus-responsive functionalities enabling enhanced therapeutic performance. A redox-sensitive polymersome functionalized with an MMP-7-activatable, masked nuclear localization signal (NLS) peptide enabled selective intracellular drug release of co-encapsulated CUR and DOX, improving tumor-selective drug delivery in pancreatic cancer cells [191]. In contrast, MMP-2-responsive PEG-PVGLIG-PLA polymersomes conjugated with the AS1411 aptamer combined enzyme-triggered release with nucleolin-mediated targeting, resulting in higher encapsulation efficiency and significantly greater tumor growth inhibition of SN38 in colorectal cancer models [192]. These findings highlight that combining protease-responsive activation with active targeting strategies can further enhance tumor selectivity and therapeutic outcomes.

Most recently, pH/cathepsin B sequential responsive nanoparticles (PSRNs) were constructed from a diblock copolymer, PEG-b-P(EPA-r-PROTAC), in which a VHL-based CDK4/6-targeting PROTAC was linked via a cathepsin B-cleavable tetrapeptide (GFLG) to the PEG-b-P(EPA) polymer. This dual-functional design maintained nanoparticle stability in circulation, enabled dissociation in the acidic tumor microenvironment, and triggered intracellular PROTAC release, enhancing cellular uptake, target protein degradation, and antitumor efficacy, while also offering potential for synergistic combination with immune checkpoint inhibitors in colorectal cancer therapy [193].

### 4.3. Triple-Stimuli-Responsive Peptide-Functionalized Nanocarriers

Building on these TME-responsive peptides, more advanced designs include triple-responsive nanoparticles and Boolean logic prodrug nanoparticles (BLPNs) that demonstrate further refinement in stimulus-specific drug release. An example of a triple-responsive nanoparticles includes a PEG-peptide (CSSSGPLGIAGQSSSK)-poly(ω-pentadecalactone-co-N-methyldiethyleneamine-co-3,3′-thiodipropionate) system loaded with anticancer drug sorafenib (SFR) and photosensitizer chlorin e6 (Ce6). This nanocarrier responds to extracellular MMP-2, acidic endosomal pH, and elevated intracellular ROS, allowing precise PEG shedding, deep tumor penetration, and accelerated intracellular drug release, resulting in enhanced cytotoxicity and tumor shrinkage in A549 xenograft models [194]. In comparison, BLPNs constructed from diblock copolymers mPEG113-GG-b-P(DPA50-r-PPa5) and mPEG113-GG-b-P(DPA50-r-NLG9195) utilizes MMP-2/9-cleavable GG (GALGLPG) peptides alongside acid-sensitive DPA2-(diisopropylamino) ethyl methacrylate and glutathione GSH-cleavable disulfide bonds. This allows for multi-stimuli intracellular activation, achieving tumor-specific accumulation of two polymeric prodrugs pheophorbide a (PPa) and NLG919, resulting in immunogenic cell death, dendritic cell maturation, and robust CD8+ T cell infiltration [195].

Similarly, MMP-2-responsive polymeric nanocapsules engineered using methacrylic acid (MMA) and zwitterionic 2-methacryloyloxyethyl phosphorylcholine (MPC) could co-deliver granzyme B (GrB) and perforin (PFN), where the proteins were conjugated and crosslinked through MMP-2-cleavable peptide sequences (CVPLGVRTK and VPLGVRTK). This peptide-mediated cleavage enabled tumor-specific protein release, resulting in enhanced intracellular delivery, increased apoptosis, and significant tumor growth inhibition with improved therapeutic efficacy compared to free proteins in 4T1 tumor-bearing mice [196].

In comparison to protease-responsive systems, pH-responsive peptide-functionalized nanoparticles exploit tumor acidity to enhance tumor selectivity and intracellular delivery. ATRAM-functionalized, albumin-coated PLGA nanoparticles loaded with doxorubicin-triphenylphosphonium (DOX-TPP) demonstrated improved tumor targeting, enhanced intracellular drug accumulation, and reduced off-target toxicity by leveraging acidity-triggered membrane interactions within the tumor microenvironment [197]. Collectively, these studies demonstrate that peptide-functionalized, stimulus-responsive polymeric nanocarriers enable precise spatial and temporal control over drug release, enhance tumor accumulation and cellular internalization, and significantly improve therapeutic efficacy compared to conventional non-responsive nanocarriers. A summary of TME-responsive peptide-functionalized polymeric nanocarriers and their stimulus-triggered drug delivery performance is provided in Table 4.

## 5. Dual-Functional and Multi-Peptide-Functionalized Polymeric Nanocarriers

Recent advances in peptide-functionalized nanocarriers have focused on integrating multiple peptide functionalities within a single polymeric system to simultaneously achieve tumor targeting, enhanced cellular internalization, and stimulus-responsive drug release. Such dual-functional and multi-peptide-functionalized polymeric nanocarriers are designed to overcome multiple biological barriers and improve therapeutic precision and efficacy.

For instance, lipid–polymer hybrid nanoparticles composed of a PLGA core and mPEG-DSPE shell were functionalized with both a pH-responsive “H” peptide and EGFR-targeting “R” peptide to enhance the co-delivery of afatinib and miR-139. This dual-peptide system effectively inhibited EGFR/HER signaling, epithelial–mesenchymal transition (EMT), and drug-resistance pathways in CRC models, resulting in enhanced apoptosis and minimal off-target toxicity [198]. Similarly, a pH-responsive, nucleus-targeted micellar nanoplatform was developed featuring a hydrophobic core composed of 5-(4-carboxyphenyl)-10,15,20-triphenylporphyrin (Por), which served both as a photosensitizer and carrier for GNA002, and a hydrophilic shell consisting of cRGD-PEG linked to hexaarginine (R6) via an acid-labile hydrazone bond. In this design, the cRGD peptide mediated integrin-specific tumor targeting, while acidic lysosomal conditions triggered hydrazone bond cleavage, exposing the R6 peptide to promote nuclear penetration. This sequential activation enhanced tumor localization, intracellular trafficking, and synergistic anticancer efficacy through the combined chemotherapy and photodynamic therapy [199].

Several dual-functional peptide strategies have been developed to overcome the multiple biological barriers associated with glioma therapy, including limited BBB transport, poor tumor penetration, and inefficient intracellular delivery. PEG-PCL micelles co-modified with bombesin and the cell-penetrating TAT peptide enabled GRPR-mediated tumor targeting and enhanced intracellular delivery of CPT. Bombesin facilitated receptor-specific targeting, while TAT promoted cellular internalization via micropinocytosis, resulting in increased tumor accumulation and improved cytotoxic efficacy in C6 glioma models [200]. PEG-PCL nanoparticles were also co-functionalized with Angiopep-2 and an activatable cell-penetrating peptide (ACP) containing an MMP-2-cleavable E8–PLGLAG–R8 sequence. Angiopep-2 mediated LRP1-dependent transport across the BBB and glioma targeting, whereas MMP-2 cleavage exposed the R8 peptide to enhance cellular uptake and tumor penetration, leading to improved glioma localization and DTX delivery compared with single-ligand formulations [201]. Building on this strategy, tandem PEG-b-poly(dithiolane trimethylene carbonate-co-ε-caprolactone) (PEG-P(DTC-co-CL)) nanomicelles were co-functionalized with Angiopep-2 and TAT to achieve sequential targeting. In this design, Angiopep-2-mediated LRP1-targeting was followed by PEG-shielded TAT peptide-mediated cellular uptake and deep tumor penetration resulting in significantly improved BBB permeation, glioma-specific DTX delivery, and antitumor efficacy with minimal systemic toxicity in an orthotopic glioma model [202]. Hu et al. conjugated the nucleolin-targeting F3 peptide (CKDEPQRRSARLSAKPAPPKPEPKPKKPKKAPAKKK) onto paclitaxel (PTX)-loaded PEG–PLA nanoparticles and co-administered them with the neuropilin-1 (NRP-1)-targeting and tumor-penetrating peptide tLyp-1. This combined strategy significantly enhanced tumor accumulation and parenchymal penetration, resulting in prolonged survival in intracranial glioma models [203]. In contrast, integration of the tenascin-C-binding sequence (FHKHKSPALSPV) and the neuropilin-1 (NRP-1)-targeting tLyp-1 motif (CGNKRTR) into a single hybrid Ft peptide, followed by conjugation to PEG-PLA nanoparticles, provided a more coordinated targeting strategy. Paclitaxel-loaded Ft nanoparticles showed greater cellular uptake in U87 and HUVEC cells, enhanced tumor localization, superior cytotoxicity and prolonged in glioma-bearing mice relative to single-targeted or non-targeted formulations, highlighting the therapeutic advantage of targeting synergistic extracellular matrix components (ECM) and receptor targeting [204]. Likewise, supramolecular aggregation-induced emission (AIE) dots based on MMP-2 sensitive PEG-peptide assemblies (PEG2000-R8-PLGLAG-EK6) and GEM-conjugated α-cyclodextrins derivatives employed EK6 peptides to prolong systemic circulation whereas MMP-2-triggered cleavage exposed the R8 segment to facilitate tumor cell internalization, followed by reductive GEM release for effective tumor inhibition in pancreatic cancer models [205].

These findings demonstrate that integrating multiple targeting motifs within a single peptide–polymer system may provide a more efficient and coordinated targeting strategy than separate peptide administration. A summary of multifunctional peptide-functionalized polymeric nanocarriers discussed in this section is presented in Table 5.

## 6. Therapeutic Peptide-Functionalized Polymeric Nanocarriers

The clinical translation of anticancer peptides is often limited by proteolytic degradation, rapid systemic clearance, poor pharmacokinetics, and inefficient intracellular delivery, necessitating the development of suitable delivery systems [206]. Accordingly, incorporation of bioactive anticancer peptides into polymeric nanocarriers has emerged as an effective strategy to protect peptides from proteolytic degradation, improve tumor-specific delivery, and preserve their biological activity during systemic circulation and intracellular delivery, thereby enhancing therapeutic efficacy [206,207,208]. Different polymer architectures influence peptide stability, intracellular delivery, and cytotoxic performance.

**Figure 3:**
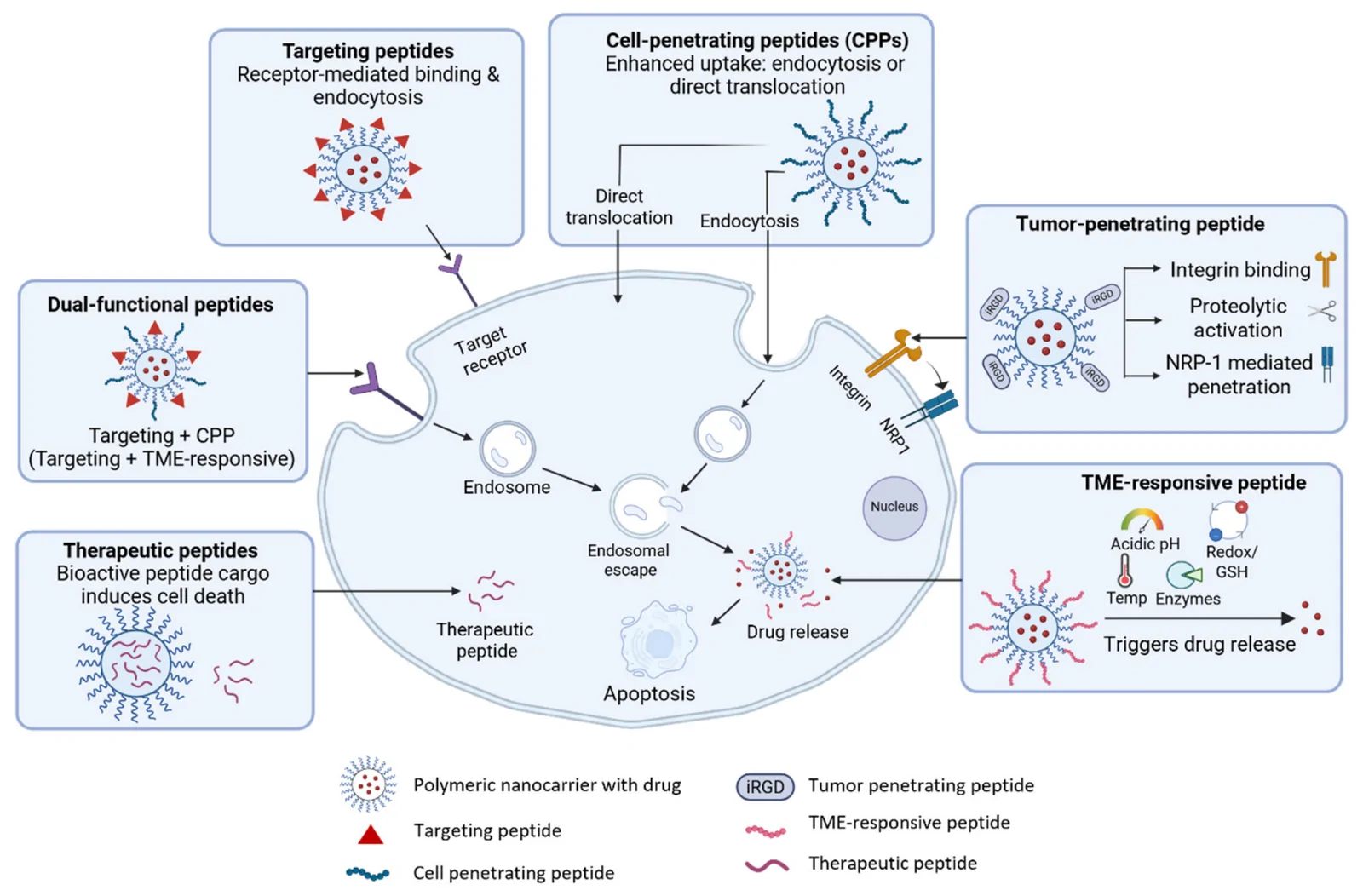
Mechanisms of action of peptide functionalities in polymeric nanocarriers. Created in BioRender. K, D. (2026) https://BioRender.com/p7vbf8q.

For example, amphiphilic hyperbranched poly(β-thioester)-PEG conjugates were used to deliver the proapoptotic KLAKLAK_2_ peptide, forming structurally stable nanoparticles with improved loading efficiency and enhanced cytotoxicity in HeLa cells, particularly when co-delivered with DOX [209]. In contrast, a virus-inspired polymer for endosomal release (VIPER) system employed an ultra-pH-sensitive PEG-based copolymer composed of 2-diisopropylaminoethyl methacrylate (DIPAMA), and pyridyl disulfide ethyl methacrylate (PDSEMA) for conjugation of D-melittin, enabling pH-triggered peptide activation following endosomal acidification. This design improved intracellular release, reduced systemic toxicity, and enhanced antitumor efficacy in vivo [210]. A non-RGD-based α_v_β_3_ integrin targeting system was developed using collagen-IV-derived peptide AXT050 which functions dually as a targeting ligand and a bioactive therapeutic agent. When conjugated to PLGA-PEG nanoparticles, this system demonstrated enhanced cellular uptake, cytotoxicity, tumor accumulation, and anti-angiogenic effects in breast cancer models [211]. A schematic overview summarizing the various peptide functionalities in polymeric nanocarriers discussed in this review, along with their functional roles and mechanisms of action, is presented in Figure 3.

## 7. Clinical Translation

Despite rapid expansion of peptide-functionalized polymeric nanocarriers at the preclinical level, translation to human oncology remains exceptionally limited. Recent studies demonstrate increasingly sophisticated approaches involving receptor targeting, tumor penetration, multivalent ligand presentation, blood–brain barrier transport, tumor-microenvironment responsiveness, and immunomodulation; nevertheless, most systems remain at the in vitro or animal-model stage. Clinical experience with polymeric peptide-containing nanomedicines is emerging, but true surface peptide-targeted polymeric nanocarriers have yet to achieve convincing clinical validation [129].

Year 03774680. is included because it represents a rare example of a polymeric nanocarrier functionalized with a peptide-based targeting ligand, although initiation predates the primary 2021–2026 review period. PRECIOUS-01 is included as a clinically translated peptide-associated polymeric nanocarrier; however, its peptides function as encapsulated tumor antigens rather than surface-targeting ligands (Table 6) [212,213].

## 8. Conclusions and Perspectives

Peptide-functionalized polymeric nanocarriers represent a versatile platform for targeted cancer therapy, combining the biological specificity of peptides with the stability and tunability of polymeric systems. Targeting peptides, cell-penetrating peptides (CPPs), and tumor-responsive sequences enhance tumor accumulation, cellular uptake, and intracellular delivery, while polymers improve pharmacokinetics, protect payloads, and enable controlled release. Multifunctional designs further address key biological barriers, resulting in improved therapeutic efficacy in preclinical models.

However, several challenges continue to limit clinical translation. Peptide instability, immunogenicity, off-target effects, and tumor heterogeneity remain critical concerns. In addition, increasing structural complexity complicates reproducibility, large-scale manufacturing, regulatory approval, and cost-effectiveness. Achieving an optimal balance between nanoparticle stability in circulation and efficient payload release at the target site remains a central design challenge.

Future progress will rely on rational design strategies supported by artificial intelligence and machine learning, enabling prediction of peptide–receptor interactions and system performance. Stimuli-responsive and activatable systems are expected to further enhance selectivity and reduce systemic toxicity. Integration with emerging modalities, including nucleic acid delivery, gene editing, and immunotherapy, will broaden the therapeutic scope. Targeting advanced disease features such as cancer stem cells and pre-metastatic niches also represents a promising direction.

Finally, successful translation will require standardized evaluation protocols, improved biocompatibility and biodegradability, and scalable manufacturing processes. Close integration of materials science, cancer biology, and translational research will be essential to bridge the gap between preclinical success and clinical application.

Overall, peptide-functionalized polymeric nanocarriers hold strong potential to advance precision oncology and enable the next generation of targeted cancer therapeutics.

## Figures and Tables

**Table 1:** Representative targeting peptide-functionalized polymeric nanocarriers for cancer therapy.

Receptor/ Target	Peptide	Peptide Sequence	Polymer	Conjugation Chemistry	Drug	Cancer Type	Ref.
TfR	T7	HAIYPRH	CBT-DC	Amide coupling	DTX + CUR	Lung	[73]
TfR	Tf	-	mPEG-PLGA	Thiol-maleimide	DTX + _siRNA (siCCAT2)	NSCLC	[74]
TfR	Tf	-	PEG–PCL	Thiol-maleimide	DOX	Glioma	[75]
TfR	TBP + Tf	CGGGHKYLRW	mPEG-P(TMC-DTC)	Thiol-maleimide/adsorption	DOX	Colorectal	[76]
CXCR4	E5	GGRSFFLLRRIQGCRFRNTVDD	DSPE-mPEG2000	Self-assembly	DOX + E5	AML	[81,82]
	E5	GGRSFFLLRRIQGCRFRNTVDD	PEG-PE	Self-assembly	DOX + E5	Solid tumors/Breast	[83,84]
CXCR4	LFC131	d-YRR(2Nal)G	CMC	Amide coupling	DTX	Lung	[85]
GRPR	Bombesin	pEQRLGNQWAVGHLM-NH2	PLGA	Amide coupling	DTX	Breast/prostate	[93,94]
GRPR	Bombesin	pEQRLGNQWAVGHLM-NH2	PEG-PPADT	Thiol-maleimide	Monomethylauristatin F	Prostate	[95]
somatostatin receptors (SSTR)	octreotide	d-FCFd-WKTCT-ol	PEG-b-PCL	Amide coupling	PTX	Breast (MCF-7)	[96]
Integrins	RGD	RGD	lecithin-PLGA	Amide coupling	PTX + Cisplatin	Lung	[105]
Integrins	RGD	RGD	PLGA–mPEG, RGD–PEG–cholesterol	-	CUR	B16 melanoma	[106]
Integrins	RGD	RGD	polydopamine-coated PHBV	Michael addition	PTX	HCC	[107]
Integrins	RGD	RGD	PEG-PCL	Amide coupling	DTX + ICG	4T1 tumor models	[108]
Integrins	cRGD	cRGD	PEG-*b*-poly(l-glutamic acid)	Thiol-maleimide	DACHPt	Glioblastoma (U87MG)	[110]
Integrins	cRGDfK	cRGDfK	PLGA	Amide coupling	GEM	Ovarian (SKOV-3)	[111]
Integrins	cRGD	cRGD	PEG-SS-PCL	Amide coupling	DOX	U87MG glioma	[112]
Integrins	cRGD	cRGD	lipid(egg phosphatidylcholine, PEGylated lipids)-PEI	Hydrophobic interactions	Survivin siRNA	Hepatocarcinoma	[113]
Integrins	PR_b	KSSPHSRN(SG)5RGDSP	PEO-PBD	Amide coupling	TNF-α	Prostate	[114]
IL-4	AP peptide	CRKRLDRN	PEG-PLA/mPEG-PAE	Thiol-maleimide	DOX	MDA-MB-231	[86]
CD44	-	CFNLPLPSRPLLR	PEG-*b*-HES-*b*-PLA	Esterification	Emodin	MDA-MB-231	[116]
CD44	A6 peptide	AC-KPSSPPEE-NH2	PCL-PEG	Amide coupling	CUR	MDA-MB-231	[117]
EGFR	GE11	CGGGSYHWYGYTPQNVI	PLGA-DSPE-PEG-Mal	Thiol-maleimide	Salinomycin-	Osteosarcoma	[118]
EGFR	GE11	CGGGSYHWYGYTPQNVI	PLGA–PEG	Thiol-maleimide	DTX + CUR	Prostate	[119]
AChR	RVG peptide	YTIWMPENPRPGTPCDIFTNSRGKRASNGGGGC	PLGA core-lipids (HSPC and DSPE)	Thiol-maleimide	PTX	Glioma	[120]
HER2	Anti-HER2 affibody peptide	VDNKFNKEMRNAYWEIALLPNLNNQQKRAFIRSLYDDPSQSANLLAEAKKLNDAQAPK	PHPMA-PAPMA	Thiol-maleimide	PTX	Breast	[121]
NRP-1	iNGRt	-	PCL	Hydrophobic interactions	DTX	Breast	[122]
MCT1	CPK	CPKSNNGVC	PLGA	Thiol-maleimide	PTX	CRC	[123]
Nucleolin	F3	CKDEPQRRSARLSAKPAPPKPEPKPKKPKKAPAKKK	PAA	Thiol-maleimide	Cisplatin	Ovarian	[124]
Angiogenic vessels	APRPG	APRPG	PEG–PLGA	Amide coupling	PTX	EA. hy926/A549	[125]
VEGFR-2 (KDR/Flk-1)	K237	HTMYYHHYQHHL	PEG–PLA	Reductive amination	PTX	Breast	[126]

**Table 2:** Representative cell-penetrating peptide-functionalized polymeric nanocarriers.

10	Peptide Sequence	Polymer	Conjugation Chemistry	Drug	Cancer Type	Ref.
Poly(L-histidine)	Poly(L-histidine)	PBAE	Electrostatic coating/self-assembly	pDNA	Melanoma	[142]
Melittin	GIGAVLKVLTTGLPALISWIKRKRQQ	Poly(OEGMA-co-DMAEMA)-b-poly(DIPAMA-co-PDSEMA) (VIPER)	Disulfide exchange	pDNA/siRNA	Cervical, Lung cancer	[144]
TAT	YGRKKRRQRRR	PLGA	Thiol-maleimide	PTX	Lung cancer	[145]
TAT	YGRKKRRQRRR	PMLA-PEI and PEG-DMMA	Thiol-maleimide	DOX	A549 cells and xenograft models	[146]
TAT	YGRKKRRQRRR	PEGylated polyphosphoesters	Amide coupling	Ce6 + TPZ	Breast cancer (MCF-7)	[147]
TAT	YGRKKRRQRRR	PLA-b-PEG-b- poly(l-histidine)- TAT and poly(l-histidine) -b-PEG	Thiol-maleimide	DOX	Different drug-sensitive and -resistant tumor models	[148]
TAT	YGRKKRRQRRR	PEG-PCL	Thiol-maleimide	CPT	Breast	[149]
TAT	YGRKKRRQRRR	PEG-PCL	Disulfide exchange	SiRNA + CPT	C6 glioma	[150]
PArg	RRRRRRRR	Hyperbranched hPDPA + β-cyclodextrin-PEG	Disulfide exchange	DOX	HeLa cells	[154]
PArg	RRRRRRRR	Hyperbranched hPDPA + β-cyclodextrin-PEG	Disulfide exchange	β-Lapachone	HeLa cells	[155]
PArg	RRRRRRRR	PEG-PAMAM dendrimers + vitamin E succinate	Amide coupling	PTX	Lung (A549), Melanoma (B16F10)	[156]
WTAS	PLKTPGKKKKGKPG- KRKEQEKKKRRTR	PBAE	Amide	Plasmid	GL-26	[158]
SCPP	RLWMRWYSPRTRAYGC	PEG-P(TMC-DTC)-PEI	Thiol-maleimide	MTX	Lung cancer	[161]
Penetratin	RQIKIWFQNRRMKWKKGG	Chitosan	Amide coupling	pDNA	HeLa cells	[162]
Angiopep-2	TFFYGGSRGKRNNFKTEEY	PEG–PCL	Thiol-maleimide	PTX	U87 MG glioma	[163]

**Table 3:** Representative iRGD (CRGDKGPDC)-functionalized polymeric nanocarriers.

Polymer	Conjugation Chemistry	Drug	Cancer Type	Ref.
CPT-PEG	Amide coupling	CPT+ IR780	Glioma	[173]
POEGMA-PDPA	Thiol-maleimide	PTX	Gastric and colon tumors	[174]
PLGA and lipids (egg phosphatidylcholine, cholesterol, and PEG–DSPE)	Thiol-maleimide	PTX + tetrandrine	Ovarian	[175]
PEG b-poly(carbonate)	Click chemistry	SCH772984 + GEM	Pancreatic cancer	[176]
PEG-b-poly(carbonate)	Click chemistry	GEM + GDC-0449	Pancreatic cancer	[177]

**Table 4:** Representative tumor microenvironment (TME)-responsive peptide-functionalized polymeric nanocarriers.

Stimulus/ Trigger	Stimulus-Responsive Peptide	Peptide Sequence	Polymer	Conjugation Chemistry	Drug(s)	Type of Cancer	Ref.
MMP	GPLGVRGDG	GPLGVRGDG	PEG-GPLGVRGDG-PDLLA	Peptide-initiated ROP	PTX	4T1 cells & H22 tumor models	[183]
MMP-2	PLGVRK	PLGVRK	PEG- PLGVRK -Cholesterol	Amide coupling	CAB	Prostate tumors	[184]
MMP	GPLGVRG	GPLGVRG	PEG-peptide-PCL, PGPMA		Colchicine and Marimastat	HT1080 human fibrosarcoma cells & 4T1 murine breast cancer orthotopic tumor model	[185]
MMP-2/9	GK8	GPVGLIGK	mPEG2K-GK8-α-TOS	Amide coupling	DTX	HT1080 cells	[186]
Cathepsin K		GGP-Nle	HPMA copolymer	DCC-mediated coupling to ALN followed by polymerization	Alendronate and TNP-470	Osteosarcoma	[187]
Cathepsin B	GFLG	GFLG	PEGylated dendrimer- GFLG- GEM	Click chemistry	GEM	4T1 murine breast cancer	[188]
Cathepsin B	GFLG	GFLG	Poly (OEGMA)	Monomer conjugation followed by polymerization	PTX and chlorin e6	Bladder cancer T24 cells and PDX models	[189]
Cathepsin B	GFLG	GFLG	Poly (2-lactobionamidoethyl methacrylamide)		PTX and capivasertib	gastric cancer models	[190]
MMP-7	NLS- MMP-7 cleavage site + -masking domain	PKKKRKV-EEEE-GPMGIAGQ	PEG-PLA	Click chemistry	DOX and CUR	Pancreatic	[191]
MMP-2		PVGLIG	PEG-PVGLIG- PLA- AS1411 aptamer	Thiol-maleimide coupling & Amide coupling	SN38	Colorectal	[192]
pH/cathepsin B	GFLG	GFLG	PEG-b-P(EPA-r-PROTAC)	Monomer conjugation followed by polymerization	PROTAC	CRC	[193]
pH, ROS and MMP-2 triple-responsive	CSSSGPLGIAGQSSSK	CSSSGPLGIAGQSSSK	PEG- CSSSGPLGIAGQSSSK-poly(ω-pentadecalactone-co-N-methyldiethyleneamine-co-3,3′-thiodipropionate)	Thiol-maleimide coupling & Amide coupling	SFR & chlorin e6 (Ce6)	A549 cells & xenograft tumor	[194]
MMP-2/9 and acid-sensitive	GG	GALGLPG	mPEG113-GG-b-P(DPA50-r-PPa5) and mPEG113-GG-b-P(DPA50-r-NLG9195)	Amide coupling	pheophorbide a (PPa) and NLG919	CT26 CRC cells & murine tumor models	[195]
MMP-2		CVPLGVRTK and VPLGVRTK	MMA and MPC	Disulfide exchange	granzyme B (GrB) and perforin (PFN)	4T1 cells and tumor models	[196]
pH	ATRAM-	GLAGLAGLLGLEGLLGLPLGLLEGLWLGLELEGN	Albumin-coated PLGA	Amide coupling	DOX–TPP	MCF-7, 4T1 tumor-bearing mice	[197]

**Table 5:** Representative dual-functional and multi-peptide-functionalized polymeric nanocarriers.

Peptide Functionality				Polymer	Drugs	Cancer Type	Ref.
Targeting Peptide	Cell-Penetrating Peptide	Enzyme-Responsive Peptide	pH-Responsive Peptide				
EGFR-targeting “R” (RPARPAR)	-	-	“H” peptide(RRpentylglycineHHHHHHHRR)	PLGA, mPEG-DSPE	Afatinib + miR-139	CRC	[198]
cRGD	Hexaarginine (R6)	-	-	Por, cRGD-PEG	Por + GNA002	HeLa, HN6, A375, MCF-7, and HN30 cancer cells	[199]
Bombesin	TAT	-	-	PEG-PCL	CPT	C6 glioma	[200]
Angiopep-2	R8 shielded by E8	MMP-2-sensitive PLGLAG	-	PEG–PCL	DTX	Glioma	[201]
Angiopep-2	TAT	-	-	PEG–P(DTC-co-CL)	DTX	Glioma	[202]
Nucleolin binding F3 peptide (CKDEPQRRSARLSAKPAPPKPEPKPKKPKKAPAKKK)	tLyp-1 (CGNKRTR) also targets NRP1	-	-	PEG-PLA	PTX	C6 glioma	[203]
tenascin C binding FHKHKSPALSPV	tLyp-1 (CGNKRTR) also targets neuropilin-1 (NRP1)			PEG-PLA	PTX	GBM	[204]
-	RRRRRRRR (R8)	MMP-2-sensitive PLGLAG	-	PEG2000, Cyclodextrin, zwitterionic stealth sequence EKEKEKEKEKEK (EK6)	GEM	Pancreatic	[205]

**Table 6:** Clinical translation of peptide-associated polymeric nanocarriers.

Year	Clinical Candidate/ Trial	Peptide	Role of Peptide	Polymeric Carrier	Therapeutic Cargo	Cancer Indication	Clinical Stage	Comment
2020	Octreotide-decorated cetuximab nanoparticles; NCT03774680	Octreotide	Surface targeting ligand; somatostatin receptor targeting	Ethylcellulose nanoparticles	Cetuximab	Colorectal cancer	Phase I	Best example of a true peptide-functionalized polymeric nanocarrier; trial record remains incomplete/outdated
2021	PRECIOUS-01; NCT04751786	NY-ESO-1-derived peptides	Encapsulated tumor antigens, not targeting ligands	PLGA nanoparticles	NY-ESO-1 peptides + IMM60 iNKT agonist	NY-ESO-1-positive solid tumors	Phase I	Important recent polymeric peptide nanomedicine, but does not meet the strict definition of surface peptide functionalization

## Data Availability

No new data were created or analyzed in this study. Data sharing is not applicable to this article.

## Abbreviations

The following abbreviations are used in this manuscript:

AChR	Nicotinic acetylcholine receptors
BBB	Blood–brain barrier
CBT-DC	Chitosan/4-hydroxyphenylboronic acid pinacol ester-based nanoparticles
CDDP	Cisplatin
CPPs	Cell-penetrating peptides
CPT	Camptothecin
CRC	Colorectal cancer
CUR	Curcumin
DIPAMA	2-Diisopropylaminoethyl methacrylate
DMMA	2,3-Dimethylmaleic anhydride
DOX	Doxorubicin
DPA 2	(Diisopropylamino) ethyl methacrylate
DSPE	1,2-Distearoyl-sn-glycero-3-phosphoethanolamine
DTX	Docetaxel
DUPA	2-[3-(1,3-Dicarboxypropyl)ureido]pentanedioic acid
EGFR	Epidermal growth factor receptor
EMT	Epithelial–mesenchymal transition
GEM	Gemcitabine
GrB	Granzyme B
GRPR	Gastrin-releasing peptide receptor
HPMA	N-(2-Hydroxypropyl) methacrylamide
lpNPs	Lipid–polymer hybrid nanoparticles
MDR	Multidrug resistance
MMA	Methacrylic acid
mPEG-P(TMC-DTC)	Poly(ethylene glycol)-block-poly(trimethylene carbonate-co-dithiolane trimethylene carbonate)
MMP	Matrix metalloproteinase
MPC	2-Methacryloyloxyethyl phosphorylcholine
O-CMC	O-Carboxymethyl chitosan
PAA	Polyacrylamide
PAE	Poly(β-amino ester)
PDLLA	Poly(D,l-lactide)
PDSEMA	Pyridyl disulfide ethyl methacrylate
PE	Phosphatidylethanolamine
PEG	Polyethylene glycol
PEI	Polyethylenimine
PEO-PBD	Poly(ethylene oxide)-b-poly(butadiene)
PFN	Perforin
PGA	Poly(glycolic acid)
PGPMA	Poly(3-guanidinopropyl methacrylamide)
PHBV	Poly(3-hydroxybutyrate-co-3-hydroxyvalerate)
PLA	Poly(lactic acid)
PLGA	Poly(lactic-co-glycolic acid)
PMLA	Poly(β-L-malic acid)
PNIPAM	Poly(N-isopropylacrylamide)
Por	5-(4-Carboxyphenyl)-10,15,20-triphenylporphyrin
PPADT	Poly(1,4-phenyleneacetone dimethylene thioketal)
PPa	Pheophorbide
PSMA	Prostate-specific membrane antigen
PTX	Paclitaxel
hPDPA	Poly(diisopropylaminoethyl Methacrylate)
PArg	Octa-arginine peptide
POEGMA-PDPA	Poly(oligoethylene glycol methacrylate)-poly(2-(diisopropylamino)ethyl methacrylate)
ROP	Ring opening polymerization
RGD	Arg-Gly-Asp
TAT	Transactivator of transcription
TBP	Transferrin-binding peptide
TME-peptides	Tumor microenvironment-responsive peptides
Tf	Transferrin
TfR	Transferrin receptor
TNF-α	Tumor necrosis factor-α
α-TOS	α-Tocopherol succinate
